# Chimeric GB virus B genomes containing hepatitis C virus p7 are infectious in vivo^☆^

**DOI:** 10.1016/j.jhep.2008.07.020

**Published:** 2008-12

**Authors:** Stephen Griffin, Rachel Trowbridge, Pia Thommes, Nigel Parry, David Rowlands, Mark Harris, Helen Bright

**Affiliations:** 1 Institute of Molecular and Cellular Biology, Faculty of Biological Sciences and Astbury Centre for Structural Molecular Biology, University of Leeds, Leeds LS2 9JT, UK; 2 Department of Virology, GSK Medicines Research Centre, Stevenage, UK

**Keywords:** Hepatitis C virus, GB virus B, p7, Amantadine, p13, Chimeric virus

## Abstract

**Background/Aims:**

The development of new therapies for hepatitis C virus (HCV) infection has been hampered by the lack of a small animal model. GB virus B (GBV-B), which infects new world monkeys, has been proposed as a surrogate system for HCV replication. Despite their short genetic distance, however, difficulties exist when extrapolating results from GBV-B to the HCV system. One way of addressing this is the creation of chimeric GBV-B containing HCV elements.

**Methods:**

Construction and analysis of GBV-B chimeras in which the p13 ion channel was replaced by its HCV counterpart, p7.

**Results:**

Replacing all, or part of, the GBV-B p13 protein with HCV p7 resulted in viable chimeras which replicated at wild-type levels in marmosets following intra-hepatic RNA injection. Serum from one animal injected with chimeric RNA was infectious in three naïve recipients, indicating that chimeras formed fully infectious virions. Amantadine, which blocks the ion channel activity of both HCV and GBV-B proteins *in vitro*, also inhibited GBV-B replication in primary hepatocytes.

**Conclusions:**

These viruses highlight the potential for chimeric GBV-B in the development of HCV-specific therapies and will provide a means of developing HCV p7 as a therapeutic target.

## 1 Introduction

Hepatitis C virus (HCV) is estimated to infect over 170 million individuals causing severe liver disease. Therapy comprising pegylated interferon-α and ribavirin is effective in only 50% of cases [1], due to drug resistance [2]. New anti-virals have been delayed by a lack of convenient small animal models, attributable to HCV’s restricted tropism for humans and chimpanzees (*Pan troglodytes*). Chimpanzees are endangered and present ethical dilemmas to researchers. Recently developed mosaic murine/human liver models are technically challenging and lack host responses to infection [3,4].

GB virus B (GBV-B) is the closest relation to HCV [5,6]; sharing 28% amino acid identity overall. It is a hepatotropic member of the Flaviviridae and is proposed to belong within the *Hepacivirus* genus alongside HCV. GBV-B primarily causes an acute, resolving hepatitis in both tamarins (*Saguinus* sp.) and common marmosets (*Callithrix jacchus*) [7–9]. Infectious sera can be passaged in primary hepatocytes from these species [10]. GBV-B has been proposed as an HCV pathogenesis model and has been used to characterise anti-virals [11,12]. Despite their short genetic distance, however, differences exist between GBV-B and HCV [11] which could be addressed by the generation of chimeras, as in other Flaviviridae [13,14]. Viable GBV-B/HCV 5′ UTR chimeras have been reported, validating this approach [15].

HCV p7 presents an attractive anti-viral target. We demonstrated its function as an amantadine-sensitive ion channel [16–18] and others have subsequently identified alternative inhibitors [19,20]. p7 comprises two *trans*-membrane alpha helices linked by a basic loop required for both function in mammalian cells [17] and replication in chimpanzees [21]. p7 localises to ER membranes [22–24] and also post-Golgi compartments [17], consistent with a recently defined role in HCV assembly [25,26].

**Fig. 1:**
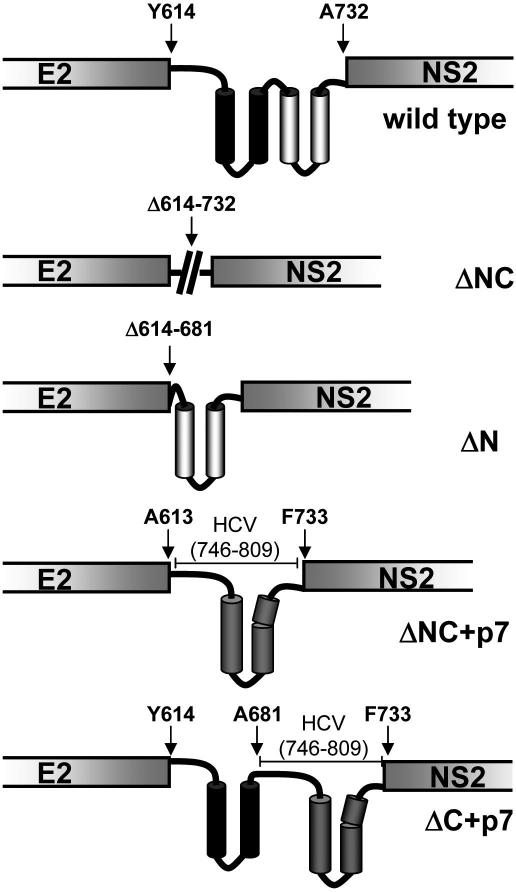
The E2–p13–NS2 region of GBV-B. p13 (614–732, GBV-B sequence) deletion mutants and insertion of HCV sequences are indicated. The N-terminal domain (614–681) in black and C-terminal domain (681–732) in white were defined according to predictions described by Ghibaudo et al. [27], HCV p7 from the J4 isolate (746–809, HCV sequence) in grey was inserted in place of either the C-terminal domain or the entire p13 region using fusion PCR.

The GBV-B p7 homologue contains four *trans*-membrane domains and is designated p13 [27]. *In vitro* analysis highlighted a second processing event between amino acids 681/682 (Fig. 1) [27] and recent *in vivo* studies have mapped this cleavage to residues 669/670; separating p13 into an N-terminal p6 and a C-terminal “p7”, the latter being required for replication in tamarins [28]. GBV-B p7 also acts as an amantadine-sensitive ion channel *in vitro* [29], confirming it as the functional equivalent of HCV p7.

Here, we describe viable chimeric GBV-B where all or part of p13 has been replaced by HCV p7, representing the first example of GBV-B/HCV coding sequence chimeras. Development of these chimeric viruses could lead to a future small animal model for p7 inhibitors.

## 2 Materials and methods

### 2.1 GBV-B/HCV DNA constructs

GBV-B (pGBB) and genotype 1b J4 HCV (pCVJ46LS) infectious clones have been described [30,31]. Deletions and chimeras were generated by overlap/fusion PCR using Pfu polymerase (Stratagene) (primer sequences and detailed methodology available upon request). Clones were verified by dsDNA sequencing. RNA was prepared using the T7 Megascript kit (Ambion) and diluted in PBS to 1 μg/μl prior to injection.

p13/p7 expression constructs were generated by PCR using wild-type or mutated sub-clones as templates (primer sequences available upon request). Amplimers (coding for amino acids S573 to S780) were cloned into pCDNA3.1 (Invitrogen) and verified by dsDNA sequencing. pCDNA-SPp7 has been described [23].

### 2.2 Human cell culture and transfection

Human embryonic kidney (HEK) 293T cells were passaged and transfected as described [23].

### 2.3 Virus infection and inhibition assays

Primary hepatocytes were isolated from euthanised marmosets using the standard two step collagenase procedure [10]. Hepatocytes were infected for 2 h with GBV-B infectious serum diluted in serum-free media (8 × 10^7^ genome copies in 0.5 ml) ± inhibitors (4 μM), then washed 3 times prior to addition of serum-free media containing drugs. Cultures were incubated for 7 days with media replaced every 2–3 days. Supernatants were stored at −20 °C for analysis. For fluorescence studies, infected cells were washed 3 times with PBS and fixed with 4% paraformaldehyde.

Vero cells in a 60 mm Petri dish were infected with 80–100 p.f.u. yellow fever virus (YFV, 17D strain) and cultured ± inhibitors (20 μM) for 6 days in a standard plaque reduction assay. Compound cytotoxicity was assessed by MTT assay in exponentially growing Vero cells over a four day period.

### 2.4 Antibodies, immunofluorescence and Western blot analysis

Immunofluorescence and western blots were carried out as described [23]. Antibody 1055 has been described [23]. Rabbit antibody 1795 was raised against peptide YPLRPVLPSQSYLQ (GBV-B amino acids 614-627), affinity-purified and used at a concentration of 1 μg/ml. Rabbit anti-GBV-B core antibody has been described [32,33].

### 2.5 Hepatic RNA injection and infection of marmosets

Marmosets (*C. jacchus*) were bred and housed at GSK. Animal use was in accordance with UK Animals (Scientific Procedures) Act 1986 and approved by internal ethical review. Animals were housed in pairs with appropriate environmental enrichment. Juvenile males and females (average 350 g) were anaesthetised with isoflurane, an incision made in the abdomen and the right lobe of the liver visualised. 150 μg of RNA (2 × 100 μl aliquots) was injected slowly into the liver at two separate sites; injections judged successful by liver blanching. Incisions were closed with internal sutures and external sub-cuticular sutures prevented wound re-opening. Appropriate analgesia was provided for 24 to 48 h post-operatively.

Marmosets were infected intravenously in the right femoral vein with 0.5 ml of GBV-B(ΔC + p7) or wild-type infectious sera (3.5 × 10^4^ or 5 × 10^5^ genome copies per animal, respectively). Weekly blood samples (0.5 ml) were taken from either the left or right femoral vein. Sera were stored at −20 °C.

### 2.6 Quantitative Analysis of GBV-B RNA

RNA was extracted from serum (50 μl), cells (10^6^) or culture supernatant (140 μl) using QIAamp viral RNA mini spin columns (Qiagen), stored at −80 °C and quantified using real-time PCR (Taqman^®^) using a core-specific primer/probe combination (available on request). Standard reactions were performed in triplicate using the EZ-RT PCR kit (Applied Biosystems) (conditions and primer sequences available on request). Quantification was achieved by standard curve using serial tenfold dilutions of *in vitro* transcribed core region RNA (from 10^8^ to 10^1^ copies/μl). For low level RNA detection, standard protocols were enhanced by performing eight replicates on at least two separate occasions to rule out false-positives.

### 2.7 Sequence analysis of chimeric GBV-B RNA

RNA was purified from marmoset AA383 liver homogenate or from animals injected with GBV-B(ΔNC + p7) RNA (five pooled serum samples, ∼750 μl, average titre 6.4 × 10^3^ genome equivalents/ml) or GBV-B(ΔC + p7) RNA (two pooled serum samples, ∼300 μl, average titre 4.8 × 10^4^ genome equivalents/ml) using the Qiagen Ultrasensitive RNeasy kit, yielding 50 μl RNA.

cDNA was generated by Superscript III (Invitrogen) using a gene-specific RT primer and 5 μl RNA. A positive control of 7.5 or 0.75 fg GBV-B(ΔNC + p7) *in vitro* transcribed RNA was included. Two rounds of PCR were performed using High Fidelity PCR mastermix (Roche) with 5 μl template (conditions and primer sequences available on request). Products were sequenced with GBV-B-specific primers using an ABI 377 sequencer (Applied Biosystems).

## 3 Results

### 3.1 Generation of chimeric GBV-B containing HCV p7

A series of viruses was constructed where all or part of p13 had been deleted or replaced by HCV genotype 1b p7 (Fig. 1). At the time, processing at 669/670 had not been described [28], so the HCV cassette replaced amino acids 614–732 (N & C-terminal region) or 682–732 (C-terminal region only) of the GBV-B sequence based on earlier studies [27], generating GBV-B(ΔNC + p7) and GBV-B(ΔC + p7), respectively. In addition, p13 (ΔNC) and amino acids 614–682 (ΔN) deletants were generated.

### 3.2 Characterisation of chimeric p13/p7 in cell culture

Signal peptidase processing of GBV-B p13 has been demonstrated in both reticulocyte lysate and transient transfection systems [27,28]. Similarly to HCV p7 [34–37], processing in this region is delayed, resulting in the presence of precursors. In addition, internal processing of p13 has recently been shown to occur at position 669/670 *in vivo* [28] requiring confirmation of appropriate processing for chimeric proteins.

We investigated sub-cellular localisation and processing of chimeric p13/HCV p7 in mammalian cells using HCV p7-specific (1055) and GBV-B p13-specific (1795) antibodies. Plasmids encoding wild-type or chimeric sequences were generated (S573–S780), encompassing the predicted signal sequence from E2, p13 and the first *trans*-membrane domain of NS2 (V582-A766).

**Fig. 2:**
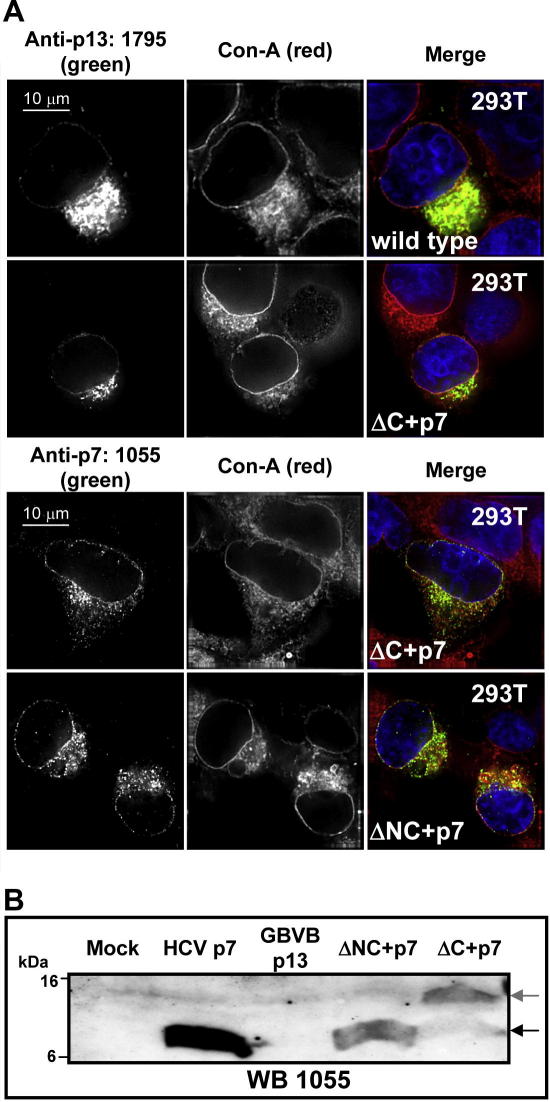
Analysis of wild-type and chimeric p13/p7 proteins in culture. Expression constructs for wild-type or chimeric p13/p7 proteins were transfected into HEK 293T cells and their intra-cellular localisation and processing by cellular signalases analysed. (A) *Top panels* – Detection of wild-type p13 and ΔC + p7 protein using 1795 (Alexa-fluor 488 nm secondary). Left column shows p13-specific fluorescence (green channel), middle column shows Alexa-fluor 594 nm-conjugated Concanavalin A, a marker for ER/Golgi membranes (red channel), and right column shows an over-lay incorporating Hoechst staining of nuclei (blue channel). *Bottom panels* – Detection of chimeric p13/p7 proteins using 1055 (Alexa-fluor 488 nm secondary) (left column), other columns as above. (B) Detection of chimeric proteins via Western-blot (WB) using anti-p7 antibody, 1055, using 10^6^ HEK 293T cells/lane lysed in 200 μl Laemmli buffer. Bands migrating at 7 kDa were identified using lysates containing HCV p7 as controls (black arrow). A higher molecular weight species corresponding to unprocessed ΔC + p7 chimeric protein was also evident (grey arrow). (For interpretation of color mentioned in this figure the reader is referred to the web version of the article.)

Immunofluorescence of HEK 293T cells expressing HCV p7, wild-type p13 or chimeric constructs revealed an ER-like staining pattern in all cases that significantly overlapped with Concanavalin A, an ER/Golgi marker (Fig. 2A). Western blots using 1055 detected p7 in the HCV positive control as well as in both chimeric constructs; albeit to lower levels (Fig. 2B). 1795 was not reactive in western blots. For the ΔNC + p7 chimera, a single reactive species co-migrated with the positive control, indicating efficient processing at the junction of the GBV-B and HCV sequence. In the case of the ΔC + p7 chimera, however, two species of ∼7 and ∼13 kDa were evident, implying partial processing reminiscent of wild-type p13.

### 3.3 GBV-B p13/HCV p7 chimeric RNAs establish productive infection in marmosets

To determine viability, naïve animals were injected with chimeric or wild-type RNA and virus replication followed for 10 weeks via serum RNA levels.

**Fig. 3:**
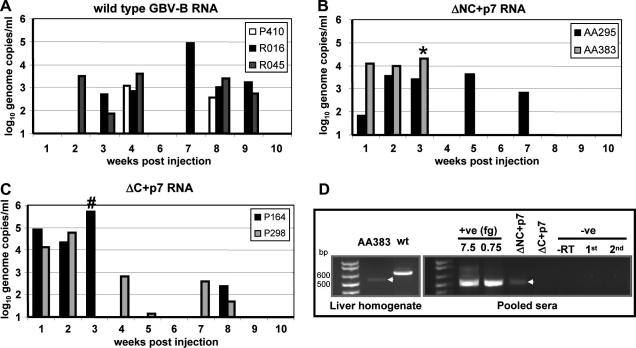
Replication of wild-type GBV-B and chimeric viruses in marmosets following intra-hepatic RNA injection. Marmosets received a total of 150 μg of either wild-type or chimeric RNAs via intra-hepatic injection. GBV-B RNA from serum was measured using quantitative RT-PCR (Taqman). (A) Titres following injection with wild-type GBV-B RNA. (B) Titres following injection with ΔNC + p7 chimeric RNA, *indicates where animal AA383 was sacrificed due to acute illness. (C) Titres following injection with ΔC + p7 RNA, ^#^indicates where serum taken from animal P164 for infection of naı¨ve animals. (D) Nested RT-PCR for p13 region on RNA extracted from animal AA383 liver homogenate (left panel) or from pooled sera of the two injected animals (right panel). A positive (+ve) control using either 7.5 or 0.75 fg of *in vitro* transcribed chimeric RNA as template is shown for comparison.

Three animals injected with wild-type RNA were PCR-positive for between three and ten weeks post-injection, although detection of RNA occurred sporadically and was of relatively low titre, ∼10^5^ genome equivalents/ml (Fig. 3A). Such sporadic replication is observed where marmosets are infected with tamarin virus [11]; pGBB being a tamarin-derived GBV-B sequence [30].

Pairs of marmosets were injected with chimeric RNAs containing HCV p7, or deletion mutants. GBV-B(ΔNC) and GBV-B(ΔN) were non-viable (data not shown), confirming p13 is necessary for GBV-B replication in marmosets and consistent with cleavage occurring between 669/670, not 681/682 [27,28]. These animals were PCR-negative at all time points, and provided baseline controls for further experiments.

ΔNC + p7 and ΔC + p7 chimeric RNAs established infection following injection and serum RNA reached wild-type levels (Fig. 3B and C) with the same sporadic pattern, although higher serum titres seemed to cluster at early time points for the chimeras and were reduced at later time points in comparison to wild-type virus. This may indicate that these infections were more rapidly resolved, though direct comparisons using small numbers of animals are difficult. One animal injected with ΔNC + p7 RNA (AA383) was sacrificed after three weeks due to acute illness. Post-mortem examination revealed hepatomegaly, and histology indicated severe hepatitis (data not shown); bacterial infection was ruled out, implying that the hepatitis was virus-mediated.

### 3.4 Chimeric virus sequences are stably maintained

Given that insertion of foreign genetic material into the GBV-B genome can cause genetic instability [38], it was important to confirm that viruses establishing productive infection maintained the chimeric sequence. Nested RT-PCR amplification of the p13 region was performed on RNA extracted from excised liver tissue of animal AA383 and wild-type GBV-B infected tamarin serum as a positive control. Reassuringly, the amplimer obtained from AA383 was smaller (505 bp) than wild-type (670 bp), as expected from the ΔNC + p7 chimera (Fig. 3D). Sequence analysis of the amplimer confirmed the presence of the HCV p7 cassette with no mutations or deletions (data not shown). Pooled sera from AA383 and AA295 gave an identical PCR amplimer which was also verified by sequencing (data not shown) and co-migrated with positive controls (Fig. 3D, right hand panels). Nested RT-PCR from ΔC + p7 animals did not yield sufficient product for sequencing.

### 3.5 Serum from chimeric RNA-injected marmosets is infectious

**Fig. 4:**
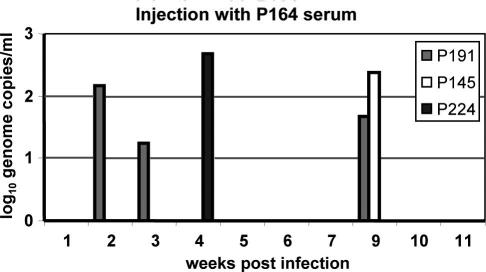
Chimeric GBV-B/HCVp7 is transmissible to naïve marmosets. Three naı¨ve marmosets were injected intra-femorally with serum from animal P164 containing 3.5 × 10^4^ copies ΔC + p7 RNA. Serum titres were monitored via Taqman RT-PCR for GBV-B RNA for 11 weeks.

To verify that the chimeric viruses generated infectious virions, serum taken from one ΔC + p7 animal (P164) at peak viraemia (Fig. 3C) was injected intravenously into three naïve animals. All animals became infected, albeit to low levels, exhibiting sporadic peaks of virus RNA in serum (Fig. 4). This low level of replication may reflect the low titre of virus inoculum, which was less than a 50% marmoset infectious dose (MID_50_) (10^6^ genome copies for wild-type tamarin-derived virus). Infection with a lower titre inoculum results either in no replication, or in sporadic replication as observed here [11]. Nevertheless, the fact that chimeric RNA was reproducibly detectable in serum up to 9 weeks post-injection showed that the virus was able to establish a low-grade infection in these animals.

### 3.6 Amantadine inhibits spread of GBV-B infection in primary marmoset hepatocytes

**Fig. 5:**
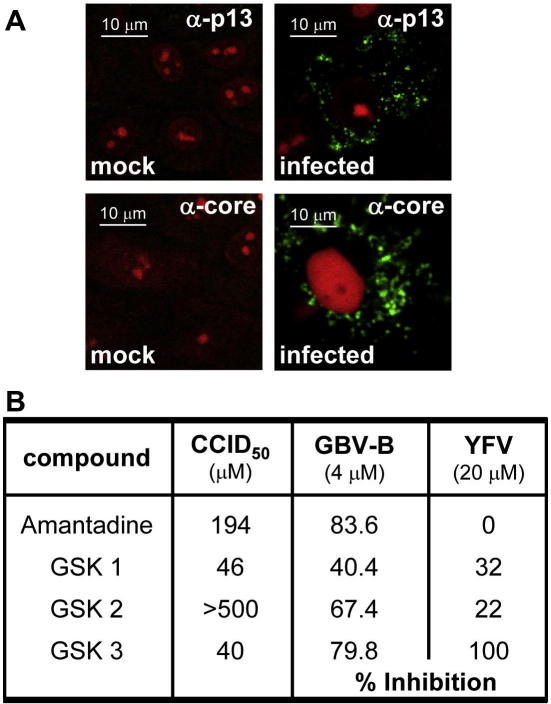
Analysis of GBV-B p13 in cultured primary marmoset hepatocytes. (A) Freshly explanted primary marmoset hepatocytes were infected with GBV-B at 8 × 10^7^ genome copies/ml in serum-free media. After 7 days cells were fixed and stained for either p13 or core protein as described in materials and methods and visualized by Confocal microscopy. (B) Replicate GBV-B infections were performed as above but in the presence of amantadine (4 μM), GSK inhibitors 1–3, or DMSO control. GBV-B RNA in the supernatant was measured using quantitative RT-PCR (Taqman) seven days post-infection. Inhibitors at fivefold concentration were also tested for effects on YFV and the cell culture 50% inhibitory dose was also determined by MTT assay. Results in virus systems are expressed as % inhibition to allow direct comparison. (For interpretation of color mentioned in this figure the reader is referred to the web version of the article.)

Resources prevented testing potential p7 inhibitors in live animals. As GBV-B readily infects primary hepatocytes [10], we utilised this system to demonstrate whether virus spread could be blocked by amantadine, which has been shown to inhibit the activity of both the C-terminal domain of p13 and HCV p7 *in vitro*[16–18,29]. To demonstrate productive infection with GBV-B, cells infected in parallel were analysed for both p13 and core protein by immunofluorescence (Fig. 5A). p13 staining displayed a punctate localisation in these cells, reminiscent of N-terminally tagged HCV p7 [23]. Core protein also showed punctate staining, likely due to lipid droplet association [32,33].

Others have found amantadine not to affect the spread of GBV-B in primary cell culture [29], so to ensure specificity we also tested three amantadine-like compounds previously shown to block the activity of HCV p7 *in vitro*; termed GSK 1–3 [39]. Compounds were also tested at a fivefold higher concentration against the related Flavivirus, YFV, which utilises similar cellular pathways to HCV/GBV-B during virus assembly, yet importantly does not encode a viroporin. In each case, virus replication was monitored by supernatant RNA levels using Taqman. MTT assays were also performed for each compound to determine the cell culture 50% inhibitory dose (CCID_50_).

Amantadine present throughout infection at 4 μM reduced GBV-B RNA levels by ∼84% compared with untreated controls, yet displayed no appreciable activity against YFV at five times this concentration (Fig. 5B). This could not have been due to cytotoxic effects as for GBV-B the drug was used at around 2% of the CCID_50_. GSK 1 and 3 were ruled out as having virus-specific effects due to their low CCID_50_, whereas the GSK-2 compound was almost as potent as amantadine against GBV-B. These data demonstrate that, like HCV, GBV-B is dependent on p7 activity for efficient production of infectious virus and suggest that an optimised chimeric virus system may be a suitable model for the testing of p7 inhibitors *in vivo*.

## 4 Discussion

This study describes the first viable *Hepacivirus* coding sequence chimeras. Despite recent advances in cell culture systems for HCV [40–42], drug development programmes would ideally include small animal systems prior to clinical trials. Tailoring the GBV-B system to include HCV proteins as described here, could expedite the development of new HCV therapies.

Levels of virus replication in this study were reproducible and comparable for both wild-type and chimeric RNAs. The RNA injection protocol, however, was clearly less efficient at establishing GBV-B infection compared to studies in tamarins, or where marmosets have been infected intravenously [8,9,30]. This may in part be due to the fact that the pGBB clone was derived from a tamarin. We previously noted that initial infection of marmosets with tamarin-derived virus results in low level, sporadic infection; requiring subsequent passages to obtain high serum RNA levels [11]. This would imply that adaptive mutations are responsible for such changes, unfortunately further investigations were beyond our previous remit and the amount of serum we were able to obtain during this work prohibited full genome sequencing. Nevertheless, we were able to show that, both in liver and serum, the chimeric p13/p7 sequence was maintained in these animals. It is reasonable to assume, therefore, that pGBB based chimeric viruses would, upon repeated passage, also become better adapted to the marmoset host.

Levels of chimeric virus replication in naïve animals receiving serum from injected subjects were low. We are convinced, however, that this represented true virus replication as one animal tested positive on three occasions over an 11 week period and the other two were positive four and nine weeks post-infection, respectively. This is unlikely to represent PCR error as no such signal was detectable in animals injected, so presumably receiving far greater amounts, with RNAs demonstrated to be non-viable (GBVB(ΔNC) and GBV-B(ΔN)). Furthermore, in studies of GBV-B with unstable HCV genetic insertions, viral RNA was undetectable by week three post-injection suggesting that non-replicating RNA does not persist for appreciable time periods [38]. Instead, low level sporadic infection likely reflects the low infectious dose; only 3.5 × 10^4^ genome equivalents each; several log_10_ lower than usually used in such studies and below that which reliably gives infection of marmosets using tamarin-derived virus; a single marmoset passage being inadequate to adapt tamarin GBV-B for efficient infection [11]. Development of this system for future drug development will obviously require more reliable infections, which may be addressed by the use of tamarins in preference to marmosets.

Previous attempts to generate chimeric GBV-B containing p7 from genotype 1a HCV (H77) in place of p13 were non-viable in tamarins [28]. Why this virus was non-viable when ΔNC + p7 replicated to wild-type levels in marmosets is unclear, though this may be due to the different HCV amino acid sequences used in each study. The same group has previously shown that inter-genotypic p7 HCV were non-viable in chimpanzees except where the p7 N and C termini were maintained as the parental genotype [21]. Two possible explanations for non-viable chimeric sequences, therefore, are a resultant disruption to signalase processing or a sequence-specific interaction of the p7 termini with other viral proteins. Changes in the p7 termini and flanking sequences have been shown to affect signalase cleavage [34,35], yet the chimeric proteins were processed similarly to GBV-B p13 in a model 293T system and localised to ER membranes (Fig. 2) as has been observed for HCV p7 [22–24]. Evidence from the HCV culture system implies that both p7 and NS2 undergo critical protein–protein interactions during virus assembly [25,26,43]. As the chimeras did not acquire mutations in the p7/p13 region, it is possible that these interactions are less important for GBV-B assembly, or that the 1b p7 is able to interact appropriately with GBV-B proteins. We cannot, however, rule out that compensatory mutations have occurred elsewhere in the chimeric genome, as has been observed in the HCV culture system [44]. Furthermore, it should be noted that this study was carried out using marmosets, not tamarins, raising the possibility that host cell factors might account for the viability of the chimeric virus RNAs.

Although resources did not permit this study to include anti-viral treatment in animals, the observation that amantadine reduced yield of GBV-B in primary hepatocyte culture implies that p7 inhibitors may be effective *in vivo*. This is contrary to the findings of other investigators in primary culture [29], yet is consistent with the *in vitro* efficacy of the drug against GBV-B p7 reported in the same study. The reason for this contradiction is unclear, but it may relate to the higher initial viral titres used in the current study highlighting a more subtle effect; the inhibitory effect being moderate at approximately tenfold. Interestingly, a recent report has found amantadine to be ineffective at preventing the spread of HCV intergenotypic chimeras in culture [45]. Our observations show that amantadine, as well as other p7 inhibitors identified from *in vitro* studies [18–20,39], vary in efficacy against chimeric HCVs in culture according to genotype as well as sub-type [46] and so may also be expected to vary in efficacy against GBV-B. Our observations are unlikely to be due to cytotoxic or off-target effects based on MTT and YFV assay data, implying that the reduction in GBV-B spread is solely due to a block in p7 function, thereby reducing secretion of infectious virus.

This work advances GBV-B as a model for HCV infection by successfully exchanging coding sequences between the two viruses, generating functional chimeras. It also demonstrates that the HCV and GBV-B p7 proteins are functionally equivalent *in vivo* and will potentially enable future testing of p7 inhibitors in a small animal model. Optimisation of this system by the use of tamarins may allow the generation of alternative chimeras expressing other HCV proteins of therapeutic interest.

## References

[1] Pawlotsky J.M. (2006). Therapy of hepatitis C: from empiricism to eradication. Hepatology.

[2] Pawlotsky J.M. (2003). Hepatitis C virus genetic variability: pathogenic and clinical implications. Clin Liver Dis.

[3] Mercer D.F., Schiller D.E., Elliott J.F., Douglas D.N., Hao C., Rinfret A. (2001). Hepatitis C virus replication in mice with chimeric human livers. Nat Med.

[4] Galun E., Burakova T., Ketzinel M., Lubin I., Shezen E., Kahana Y. (1995). Hepatitis C virus viremia in SCID–>BNX mouse chimera. J Infect Dis.

[5] Ohba K., Mizokami M., Lau J.Y., Orito E., Ikeo K., Gojobori T. (1996). Evolutionary relationship of hepatitis C, pesti-, flavi-, plantviruses, and newly discovered GB hepatitis agents. FEBS Lett.

[6] Muerhoff A.S., Leary T.P., Simons J.N., Pilot-Matias T.J., Dawson G.J., Erker J.C. (1995). Genomic organization of GB viruses A and B: two new members of the Flaviviridae associated with GB agent hepatitis. J Virol.

[7] Jacob J.R., Lin K.C., Tennant B.C., Mansfield K.G. (2004). GB virus B infection of the common marmoset (*Callithrix jacchus*) and associated liver pathology. J Gen Virol.

[8] Lanford R.E., Chavez D., Notvall L., Brasky K.M. (2003). Comparison of tamarins and marmosets as hosts for GBV-B infections and the effect of immunosuppression on duration of viremia. Virology.

[9] Bukh J., Apgar C.L., Govindarajan S., Purcell R.H. (2001). Host range studies of GB virus-B hepatitis agent, the closest relative of hepatitis C virus, in New World monkeys and chimpanzees. J Med Virol.

[10] Beames B., Chavez D., Guerra B., Notvall L., Brasky K.M., Lanford R.E. (2000). Development of a primary tamarin hepatocyte culture system for GB virus-B: a surrogate model for hepatitis C virus. J Virol.

[11] Bright H., Carroll A.R., Watts P.A., Fenton R.J. (2004). Development of a GB virus B marmoset model and its validation with a novel series of hepatitis C virus NS3 protease inhibitors. J Virol.

[12] Beames B., Chavez D., Lanford R.E. (2001). GB virus B as a model for hepatitis C virus. Ilar J.

[13] Pletnev A.G., Bray M., Huggins J., Lai C.J. (1992). Construction and characterization of chimeric tick-borne encephalitis/dengue type 4 viruses. Proc Natl Acad Sci USA.

[14] Chambers T.J., Nestorowicz A., Mason P.W., Rice C.M. (1999). Yellow fever/Japanese encephalitis chimeric viruses: construction and biological properties. J Virol.

[15] Rijnbrand R., Yang Y., Beales L., Bodola F., Goettge K., Cohen L. (2005). A chimeric GB virus B with 5′ nontranslated RNA sequence from hepatitis C virus causes hepatitis in tamarins. Hepatology.

[16] Clarke D., Griffin S., Beales L., Gelais C.S., Burgess S., Harris M. (2006). Evidence for the formation of a heptameric ion channel complex by the hepatitis C virus p7 protein in vitro. J Biol Chem.

[17] Griffin S.D., Harvey R., Clarke D.S., Barclay W.S., Harris M., Rowlands D.J. (2004). A conserved basic loop in hepatitis C virus p7 protein is required for amantadine-sensitive ion channel activity in mammalian cells but is dispensable for localization to mitochondria. J Gen Virol.

[18] Griffin S.D., Beales L.P., Clarke D.S., Worsfold O., Evans S.D., Jaeger J. (2003). The p7 protein of hepatitis C virus forms an ion channel that is blocked by the antiviral drug, Amantadine. FEBS Lett.

[19] Premkumar A., Wilson L., Ewart G.D., Gage P.W. (2004). Cation-selective ion channels formed by p7 of hepatitis C virus are blocked by hexamethylene amiloride. FEBS Lett.

[20] Pavlovic D., Neville D.C., Argaud O., Blumberg B., Dwek R.A., Fischer W.B. (2003). The hepatitis C virus p7 protein forms an ion channel that is inhibited by long-alkyl-chain iminosugar derivatives. Proc Natl Acad Sci USA.

[21] Sakai A., Claire M.S., Faulk K., Govindarajan S., Emerson S.U., Purcell R.H. (2003). The p7 polypeptide of hepatitis C virus is critical for infectivity and contains functionally important genotype-specific sequences. Proc Natl Acad Sci USA.

[22] Carrere-Kremer S., Montpellier-Pala C., Cocquerel L., Wychowski C., Penin F., Dubuisson J. (2002). Subcellular localization and topology of the p7 polypeptide of hepatitis C virus. J Virol.

[23] Griffin S., Clarke D., McCormick C., Rowlands D., Harris M. (2005). Signal peptide cleavage and internal targeting signals direct the hepatitis C virus p7 protein to distinct intracellular membranes. J Virol.

[24] Haqshenas G., Mackenzie J.M., Dong X., Gowans E.J. (2007). Hepatitis C virus p7 protein is localized in the endoplasmic reticulum when it is encoded by a replication-competent genome. J Gen Virol.

[25] Jones C.T., Murray C.L., Eastman D.K., Tassello J., Rice C.M. (2007). Hepatitis C virus p7 and NS2 proteins are essential for production of infectious virus. J Virol.

[26] Steinmann E., Penin F., Kallis S., Patel A.H., Bartenschlager R., Pietschmann T. (2007). Hepatitis C Virus p7 protein is crucial for assembly and release of infectious virions. PLoS Pathog.

[27] Ghibaudo D., Cohen L., Penin F., Martin A. (2004). Characterization of GB virus B polyprotein processing reveals the existence of a novel 13-kDa protein with partial homology to hepatitis C virus p7 protein. J Biol Chem.

[28] Takikawa S., Engle R.E., Emerson S.U., Purcell R.H., St Claire M., Bukh J. (2006). Functional analyses of GB virus B p13 protein: development of a recombinant GB virus B hepatitis virus with a p7 protein. Proc Natl Acad Sci USA.

[29] Premkumar A., Dong X., Haqshenas G., Gage P.W., Gowans E.J. (2006). Amantadine inhibits the function of an ion channel encoded by GB virus B, but fails to inhibit virus replication. Antiviral Ther.

[30] Bukh J., Apgar C.L., Yanagi M. (1999). Toward a surrogate model for hepatitis C virus: an infectious molecular clone of the GB virus-B hepatitis agent. Virology.

[31] Yanagi M., St Claire M., Shapiro M., Emerson S.U., Purcell R.H., Bukh J. (1998). Transcripts of a chimeric cDNA clone of hepatitis C virus genotype 1b are infectious in vivo. Virology.

[32] Hope R.G., Murphy D.J., McLauchlan J. (2002). The domains required to direct core proteins of hepatitis C virus and GB virus-B to lipid droplets share common features with plant oleosin proteins. J Biol Chem.

[33] Targett-Adams P., Schaller T., Hope G., Lanford R.E., Lemon S.M., Martin A. (2006). Signal peptide peptidase cleavage of GB virus B core protein is required for productive infection in vivo. J Biol Chem.

[34] Isherwood B.J., Patel A.H. (2005). Analysis of the processing and transmembrane topology of the E2p7 protein of hepatitis C virus. J Gen Virol.

[35] Carrere-Kremer S., Montpellier C., Lorenzo L., Brulin B., Cocquerel L., Belouzard S. (2004). Regulation of hepatitis C virus polyprotein processing by signal peptidase involves structural determinants at the p7 sequence junctions. J Biol Chem.

[36] Mizushima H., Hijikata M., Asabe S., Hirota M., Kimura K., Shimotohno K. (1994). Two hepatitis C virus glycoprotein E2 products with different C termini. J Virol.

[37] Lin C., Lindenbach B.D., Pragai B.M., McCourt D.W., Rice C.M. (1994). Processing in the hepatitis C virus E2-NS2 region: identification of p7 and two distinct E2-specific products with different C termini. J Virol.

[38] Haqshenas G., Dong X., Netter H., Torresi J., Gowans E.J. (2007). A chimeric GB virus B encoding the hepatitis C virus hypervariable region 1 is infectious in vivo. J Gen Virol.

[39] St. Gelais C., Tuthill T.J., Clarke D.S., Rowlands D.J., Harris M., Griffin S. (2007). Inhibition of hepatitis C virus p7 membrane channels in a liposome-based assay system. Antiviral Res.

[40] Lindenbach B.D., Evans M.J., Syder A.J., Wolk B., Tellinghuisen T.L., Liu C.C. (2005). Complete replication of hepatitis C virus in cell culture. Science.

[41] Wakita T., Pietschmann T., Kato T., Date T., Miyamoto M., Zhao Z. (2005). Production of infectious hepatitis C virus in tissue culture from a cloned viral genome. Nat Med.

[42] Zhong J., Gastaminza P., Cheng G., Kapadia S., Kato T., Burton D.R. (2005). Robust hepatitis C virus infection in vitro. Proc Natl Acad Sci USA.

[43] Pietschmann T., Kaul A., Koutsoudakis G., Shavinskaya A., Kallis S., Steinmann E. (2006). Construction and characterization of infectious intragenotypic and intergenotypic hepatitis C virus chimeras. Proc Natl Acad Sci USA.

[44] Yi M., Ma Y., Yates J., Lemon S.M. (2007). Compensatory mutations in E1, p7, NS2, and NS3 enhance yields of cell culture-infectious intergenotypic chimeric hepatitis C virus. J Virol.

[45] Steinmann E., Whitfield T., Kallis S., Dwek R.A., Zitzmann N., Pietschmann T. (2007). Antiviral effects of amantadine and iminosugar derivatives against hepatitis C virus. Hepatology.

[46] Griffin S, St Gelais C, Owsianka A, Patel A, Rowlands D, Harris M. Genotype-dependent sensitivity of hepatitis C virus to inhibitors of the p7 ion channel. Hepatology, 2008 in press. 10.1002/hep.22555 PMC7615706 18828153

